# Microglial responses to amyloid β peptide opsonization and indomethacin treatment

**DOI:** 10.1186/1742-2094-2-18

**Published:** 2005-08-19

**Authors:** Ronald Strohmeyer, Carl J Kovelowski, Diego Mastroeni, Brian Leonard, Andrew Grover, Joseph Rogers

**Affiliations:** 1 L.J. Roberts Center, Sun Health Research Institute, 10515 West Santa Fe Drive, Sun City, AZ 85351 USA

## Abstract

**Background:**

Recent studies have suggested that passive or active immunization with anti-amyloid β peptide (Aβ) antibodies may enhance microglial clearance of Aβ deposits from the brain. However, in a human clinical trial, several patients developed secondary inflammatory responses in brain that were sufficient to halt the study.

**Methods:**

We have used an in vitro culture system to model the responses of microglia, derived from rapid autopsies of Alzheimer's disease patients, to Aβ deposits.

**Results:**

Opsonization of the deposits with anti-Aβ IgG 6E10 enhanced microglial chemotaxis to and phagocytosis of Aβ, as well as exacerbated microglial secretion of the pro-inflammatory cytokines TNF-α and IL-6. Indomethacin, a common nonsteroidal anti-inflammatory drug (NSAID), had no effect on microglial chemotaxis or phagocytosis, but did significantly inhibit the enhanced production of IL-6 after Aβ opsonization.

**Conclusion:**

These results are consistent with well known, differential NSAID actions on immune cell functions, and suggest that concurrent NSAID administration might serve as a useful adjunct to Aβ immunization, permitting unfettered clearance of Aβ while dampening secondary, inflammation-related adverse events.

## Background

Chemotactic and phagocytic responses of microglia to amyloid β peptide (Aβ) have been inferred from postmortem autopsy evaluations [1-3], animal studies [4,5], and an in vitro model in which cultured rodent microglia were placed directly on Alzheimer's disease (AD) cortical sections [5,6]. Although these valuable experiments confirm that microglia cluster around and may help clear Aβ deposits, new questions have arisen concerning the effects of various agents on these microglial interactions with Aβ. In particular, several studies have indicated that the opsonization of Aβ deposits with anti-Aβ antibodies facilitates microglia-mediated Aβ clearance [6,7]. Here, binding of the antibodies to the Aβ target presumably enhances microglial recognition of and subsequent responses to the target through Fc receptors expressed by the microglia [6,7]. Based on these results, it has been suggested that microglial responses to Aβ might represent so beneficial an inflammatory action that anti-inflammatory drugs might actually be detrimental as a treatment for AD [8]. Alternatively, multiple epidemiologic studies [9,10] have reported decreased risk for AD in persons who take common nonsteroidal anti-inflammatory drugs (NSAIDs).

Over the last decade, our laboratory has developed reliable methods for culturing microglia from rapid (< 4 hour) brain autopsies of AD patients [11,12]. These cultures uniquely match the species, developmental stage, and disease state of AD subjects, and provide the ready experimental manipulability that is helpful in assessing complex physiologic processes such as chemotaxis, phagocytosis, secretory activity, and drug responses. In order to quantitatively assay these processes in the context of microglial interactions with Aβ, we seeded AD microglial cultures into wells containing pre-aggregated Aβ1-42 spots dried down to the well floor. Subsequent experiments measured migration of the cells to the Aβ spots, phagocytosis of the Aβ spots, pro-inflammatory cytokine secretion, and the effects on these processes when Aβ spots were opsonized with an anti-Aβ antibody or when microglia were treated with a common nonsteroidal anti-inflammatory drug (NSAID), indomethacin. Overall, opsonization with Aβ antibody enhanced microglial migration to and phagocytosis of Aβ. Indomethacin had little to no effect on these responses, but did significantly inhibit microglial secretion of IL-6.

## Methods

### AD microglia cultures

Cultures of microglia from rapid (< 4 hours) autopsies of six antemortem-evaluated, neuropathologically-confirmed AD patients were prepared using our previously published methods [11,12]. By immunoreactivity, these cultures are consistently negative for neuron, astrocyte, oligodendrocyte, and fibroblast markers, consistently positive for multiple markers of activated microglia, and readily maintained at purities of 98% or higher [11,12]. Microglia cultures from all six AD patients were used for biochemical assays. Additional cultures from one of these patients were used for quantitative evaluation of chemotaxis and phagocytosis, and additional cultures from two more of these patients were used for qualitative replication of the chemotaxis and phagocytosis results. At 3–7 days post-plating, the microglia were trypsinized and replated at 50,000 cells/well in 12-well plates. Prior to replating, 2 μl of a 1 mM solution of Aβ1-42 (Bachem) in PBS (pH 7.4) was dried down to the well floor. Each well received two such Aβ spots, and there were three wells per experimental condition, so that a total of six Aβ spots were quantified per experimental condition. Serum-free medium was used throughout the experiments. Control wells containing no Aβ or no microglia were also prepared.

### Treatment with anti-Aβ antibody

Prior to seeding with microglia, selected wells were pretreated with vehicle (medium) only or with 10 μg/ml 6E10 (Signet Laboratories), a mouse monoclonal antibody directed against the first 17 (N-terminal) amino acids in the Aβ sequence. In some experiments, a 2 μg/ml concentration of 6E10 was included in order to evaluate effects at a lower dose.

### Treatment with indomethacin

Prior to seeding with microglia, selected wells were pretreated with vehicle (medium) only or with 1.0 μg/ml indomethacin. Indomethacin, at 1.0 μg/ml, and vehicle were also replenished at Days 3, 6, and 9 in the course of medium changes. The 1.0 μg/ml indomethacin concentration is at the upper end of the physiologically normal range achieved in blood after therapeutic doses of the drug [13], and was chosen to insure that any failure of indomethacin to affect chemotaxis to or phagocytosis of Aβ was not due to inadequate drug dosage. In some experiments, a 0.1 μg/ml concentration of indomethacin, which is at the lower end of the physiologically normal range achieved in blood after therapeutic doses, was included in order to evaluate effects at a lesser concentration.

### Cytochemistry and immunocytochemistry

For qualitative evaluations of microglial responses to Aβ, microglial cultures were briefly fixed with 4% buffered paraformaldehyde, then immunoreacted overnight with 1:1000 (0.5 μg/ml) LN3 antibody (MP Biomedical) directed against the major histocompatibility complex type II cell surface glycoprotein, using our previously published methods [11,14,15]. Vectastain ABC kits (Vector Laboratories) were employed using the manufacturer's protocols to detect immunoreactivity with bright field optics. Aβ spots could be sufficiently resolved under these conditions by their modest opaqueness under bright field optics. To visualize Aβ spots in phagocytosis experiments, the wells were washed gently in distilled water (3 × for 5 min each), incubated with 0.1% Thioflavine S (Sigma) for 10 min, washed once in distilled water (5 min), then dehydrated and fixed with 4% buffered paraformaldehyde. In additional experiments, Aβ immunocytochemistry was applied in selected wells so as to graphically illustrate Aβ removal and microglial uptake of Aβ. In these studies, microglial cultures with Aβ spots were briefly fixed with 4% buffered paraformaldehyde and incubated overnight with 1:1000 (1 μg/ml) anti-Aβ antibody 4G8 (Signet Laboratories). Detection of immunoreactivity was accomplished using Vectastain ABC kits (Vector Laboratories) and the manufacturer's suggested protocols.

### Microglial migration to Aβ spots

**Figure 1:**
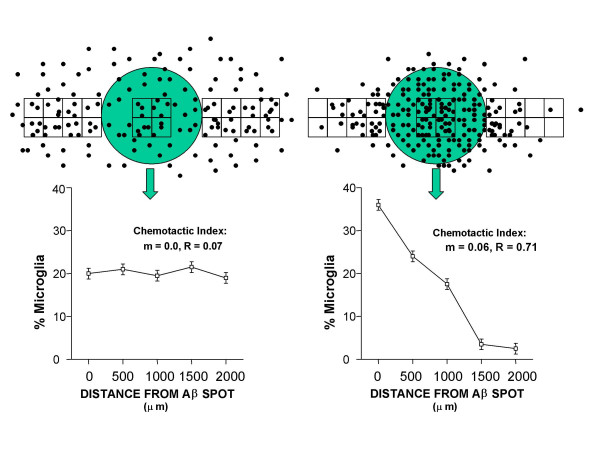
**Paradigm for estimation of microglial chemotaxis to Aβ**. Upper left panel shows a hypothetical example at Day 1, when microglia (black dots) are uniformly distributed relative to Aβ spots (gray circle). A plot of microglial density within 500 μm × 500 μm grid squares at increasing proximity to the spot (lower left) is therefore relatively flat, with a slope near 0, indicative of little or no migratory activity at this early time point. After 9 days (right panels), microglia are clustered over and around the Aβ spot, yielding a pronounced slope to the plot, consistent with chemotaxis to the Aβ. Previous studies have referred to such slopes as "chemotactic indices" [c.f., 15].

Microglial cultures were assessed on Day 3 and Day 9 after initial plating. Each Aβ spot was visualized under phase contrast optics at 100 × (10 × objective), and photomontages were made of the spot and surrounding area out to a radius of 2 mm from the spot perimeter. A grid was then placed over the photomontages. The number and percentages of microglia within four 500 μm × 500 μm (0.25 mm^2^) grid squares centered on the Aβ spot and within sets of four 500 μm × 500 μm squares at progressively greater distances from the spot were recorded. The distance intervals for the grid squares were 0, 500, 1000, 1500, and 2000 μm from the Aβ spot, and each distance interval was measured in quadruplicate (Fig. 1). A total of 141,455 microglia were individually hand-counted in this way. Chemotaxis was evaluated by changes in the distributions of microglia relative to the Aβ spots over time, with relatively flat distributions indicative of little or no chemotaxis, and increasingly negative slopes to the distributions indicative of migration toward the Aβ spots (Fig. 1). Slopes of the distributions (m) were operationally defined as the "chemotactic index" [15] for each condition, and the statistical reliability of the measures was assessed with Pearson's Product Momentum (R) statistic and with analysis of variance (ANOVA) techniques. The simplest ANOVAs assessed, for each treatment condition, significant differences in the distributions of microglia over the progressive distance intervals from the Aβ spot, with percentage of microglia at a particular distance (grid square) as the dependent variable and distance from the Aβ spot (0, 500, 1000, 1500, and 2000 μm) as the single factor. Pearson's R Statistic was then run to confirm that the alterations in microglial distributions were consistent with chemotaxis (i.e., showed a significant negative correlation with distance from Aβ) rather than some other response pattern. Dose dependence was evaluated using two-way ANOVAs, with percentage of microglia as the dependent variable, distance from the Aβ spot as the first factor, and drug dose as the second factor. Significant interactions of distance with drug dose thereby provided statistical evidence that the different drug doses differentially affected microglial distributions. A similar approach was taken for comparisons of different treatment conditions (e.g., anti-Aβ antibody exposure ± indomethacin treatment). All data collection was by a technician blind to experimental condition.

### Tests of microglial proliferation

BrdU staining kits (Zymed/Invitrogen) were applied to selected wells in order to assess whether shifts in microglial distributions over time might be due to differential proliferation of microglia relative to Aβ spots as opposed to migration of the cells. Staining with BrdU followed the manufacturer's recommended directions.

### Microglial phagocytosis of Aβ spots

At Day 12 postplating, selected wells were histochemically reacted with Thioflavine S, as described earlier, and visualized at 100 × (10 × objective) with a confocal microscope. Using the ability of the confocal microscope to optically section an object at precise distances, the number of 10 μm optical slices from the well floor to the top of the remaining Aβ spot was recorded by an investigator blinded to the experimental conditions imposed in each well. The data were then assessed statistically using 2-way ANOVAs, with spot thickness as the outcome measure, antibody treatment (vehicle only, 2 μg/ml anti-A↕ IgG, or 10 μg/ml anti-A↕ IgG) as the first factor, and NSAID treatment (vehicle only, 0.1 μg/ml indomethacin, or 1.0 μg/ml indomethacin) as the second factor.

### Microglial secretion of cytokines

To assess the effects of opsonization with anti-Aβ antibodies, microglial cultures were preincubated with vehicle or 10 μg/ml anti-Aβ monoclonal 6E10 followed by 4 hours exposure to 0 or 10 μM preaggregated Aβ1-42 (Bachem). Conditioned medium was then subjected to TNF-α ELISA (R&D Systems) using the manufacturer's protocols. To confirm the results with another pro-inflammatory cytokine, and to evaluate the interaction of indomethacin with antibody opsonization, microglial cultures were preincubated with vehicle or 10 μg/ml 6E10, as before, but in the presence or absence of 1 μg/ml indomethacin. After incubation for 4 hours with 0 or 10 μM Aβ1-42, the conditioned medium was subjected to IL-6 ELISA (R&D Systems) using the manufacturer's protocols.

## Results

### Microglial migration to Aβ spots

**Figure 2:**
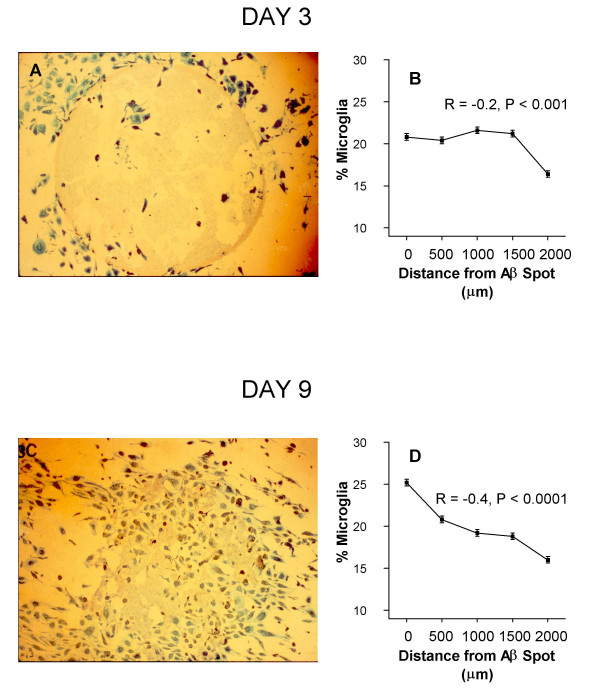
**Typical responses of cultured AD microglia to pre-aggregated Aβ1-42 spots dried down to the well floor. **A) Micrograph of Aβ spot (light brown stain) and LN3 immunoreactive microglia (blue stain) 3 days postplating (vehicle control) (4 × objective). B) Graphic summary of microglial distributions at 3 days postplating (pooled data over all conditions). C) Parallel well 9 days postplating (vehicle control) (4 × objective). Wells seeded with microglia but without Aβ spots exhibited only random distributions of cells (not shown). D) Graphic summary of microglial distributions at Day 9 (pooled data over all conditions). Similar and highly significant shifts over time were observed in all treatment conditions when Aβ spots were present (see text).

**Figure 3:**
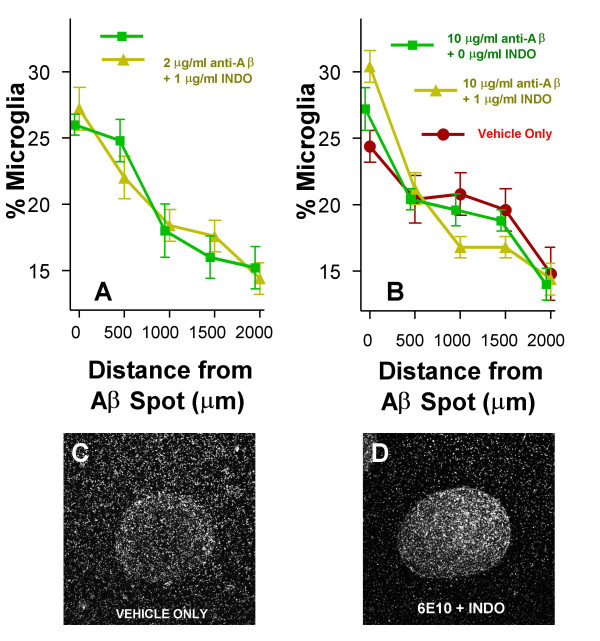
**Microglial distributions after 9 days incubation with Aβ spots**. A) Treatment with 2 μg/ml anti-Aβ antibody plus (yellow) or minus (green) 1 μg/ml indomethacin (INDO). B) Treatment with vehicle control (red) or 10 μg/ml anti-Aβ antibody plus (yellow) or minus (green) 1 μg/ml indomethacin. C) Representative phase contrast image (4 × objective) of microglia and an Aβ spot when treated with vehicle only. D) Representative phase contrast image (4 × objective) of microglia and an Aβ spot when treated with 10 μg/ml anti-Aβ antibody plus 1 μg/ml indomethacin.

Overall and within each treatment condition there were shifts in microglial distributions, consistent with chemotaxis, that were both visually apparent (Figs. 2A, 2C) and statistically significant (Figs. 2B, 2D). By Day 3, the greatest concentrations of microglia were midway between the most distal and proximal points from the Aβ spots (F_Distance _= 40.1, P = 0.000; R = -.17, P = 0.000; m = -.016) (Fig. 2B). By Day 9, the greatest concentrations of microglia were at or adjacent to the spots (F_Distance _= 99.2, P = 0.000; R = -.41, P = 0.000; m = -.041) (Fig. 2D). Microglia seeded into wells without Aβ spots essentially remained randomly distributed throughout these time periods. Opsonization with anti-Aβ antibodies significantly enhanced chemotaxis-like shifts in microglial distributions, an effect that was especially prominent at Day 9 (Table 1) (Fig. 3). Indomethacin had no significant or obvious effect on changes in microglial distributions over time under any of the Aβ antibody treatment conditions. Indeed, the largest chemotactic index (slope) observed in the study occurred at the highest dose of indomethacin (1.0 μg/ml indomethacin plus 10 μg/ml anti-Aβ) (F_Distance _= 38.9, P = 0.000; R = 0.69, P = 0.000; m = -.073), and the second largest chemotactic index occurred at the second highest dose of indomethacin (0.1 μg/ml indomethacin plus 10 μg/ml anti-Aβ) (F_Distance _= 12.9, P = 0.000; R = -.53, P = 0.000; m = -.060 (Fig. 3).

**Table 1:** Effects of opsonization with anti-Aβ antibody 6E10 on chemotaxis-like changes in microglia distributions

	**ANOVA**		**PEARSON'S**		**SLOPE**
	**F**	**P**	**R**	**P**	**m**
Day 3					
0 μg/ml anti-Aβ	3.7	0.007	-0.26	0.005	-0.022
2 μg/ml anti-Aβ	2.5	0.040	-0.14	NS	-0.023
10 μg/ml anti-Aβ	5.5	0.000	-0.27	0.003	-0.027
Dose dependence*	3.6	0.008			
Day 9					
0 μg/ml anti-Aβ	5.6	0.000	-0.37	0.000	-0.040
2 μg/ml anti-Aβ	11.2	0.000	-0.050	0.000	-0.051
10 μg/ml anti-Aβ	16.4	0.000	-0.57	0.000	-0.056
Dose dependence*	2.3	0.050			

*Dose × distance interaction term

### Differential proliferation versus chemotaxis

Proliferation of microglia more proximal to the Aβ spots, rather than true chemotaxis, did not explain the shifts in microglial distributions that were exhibited over time under the various treatment conditions. There was little to no BrdU staining under any condition (not shown) and, in fact, there was a slight but significant decrease in microglial numbers in all treatment conditions and overall from Day 3 (mean microglial density/0.25 mm^2 ^grid square = 40.8 ± 0.3) to Day 9 (mean microglial density/0.25 mm^2 ^grid square = 37.8 ± 0.4) (F_Overall _= 34.5, P = 0.000). Consistent with our previous experience, AD microglia stimulated with M-CSF as a positive control showed little to no evidence of proliferation. However, M-CSF-stimulated THP-1 cells (a monocyte line often used as a surrogate for microglia) that were run in parallel did show clear proliferation under the same BrdU assay conditions (data not shown).

### Microglial phagocytosis of Aβ

**Figure 4:**
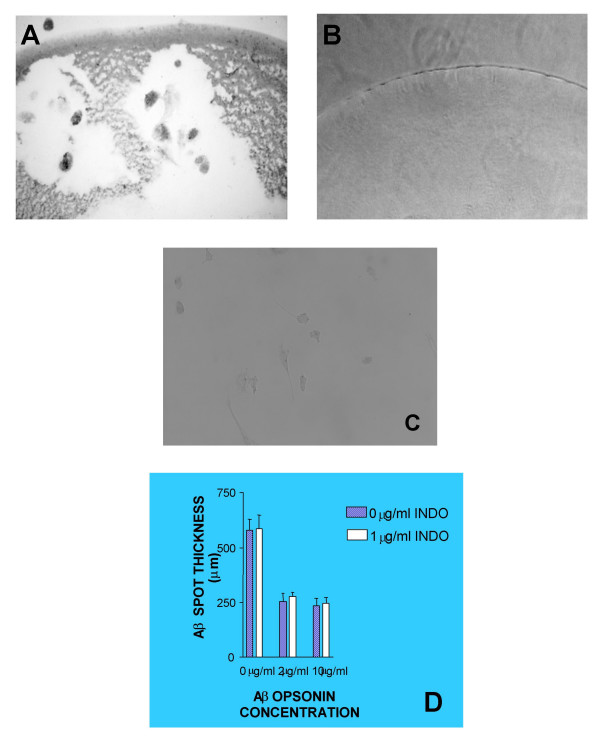
**Evidence for phagocytosis of Aβ by AD microglia in vitro under the various experimental conditions. **A) Twelve days after plating AD microglia with Aβ spots, diminution of the spots was visually apparent and microglia concurrently had become immunoreactive for Aβ even under vehicle control conditions, as shown here (anti-Aβ antibody 4G8 immunocytochemistry). B) In the absence of microglia, the Aβ spots remained visibly intact (phase contrast). C) Likewise, prior to exposure to Aβ spots the microglia exhibited little or no immunoreactivity for Aβ (anti-Aβ antibody 4G8 immunocytochemistry). D) Summary data illustrating the effects of indomethacin and 6E10 opsonization on Aβ spot thickness. Microglia in this model system carpet the top of Aβ spots (c.f., Fig. 2C) and therefore appear to clear the Aβ from the top down, resulting in progressive thinning of the spot, as measured here. With prolonged exposure, cracks and holes in the spot appear, as shown in Fig. 4A.

After incubation with microglia under the various experimental conditions, visible degradation of Aβ spots was apparent (Fig. 4A), whereas Aβ spots in wells not containing microglia remained visibly intact over the same time periods (Fig. 4B). Concurrent with degradation of the Aβ spots, microglia in contact with the spots became Aβ immunoreactive (Fig. 4A), whereas they exhibited little to no Aβ immunoreactivity prior to their being seeded into the wells (Fig. 4C). Opsonization of Aβ spots with 2 μg/ml anti-Aβ antibody 6E10 (F = 28.7, P = 0.006) or 10 μg/ml anti-Aβ antibody 6E10 (F = 35.3, P = 0.004) resulted in significantly smaller (thinner) Aβ spots compared to the vehicle control condition (Fig. 4D). These effects were not significantly or materially inhibited by indomethacin even at the highest, 1.0 μg/ml indomethacin concentration (for 2 μg/ml anti-Aβ ± 1.0 μg/ml indomethacin: F = 0.3, P = 0.639) (for 10 μg/ml anti-Aβ plus ± 1.0 μg/ml indomethacin: F = 0.9, P = 0.402) (Fig. 4D).

### Microglial secretion of cytokines

**Figure 5:**
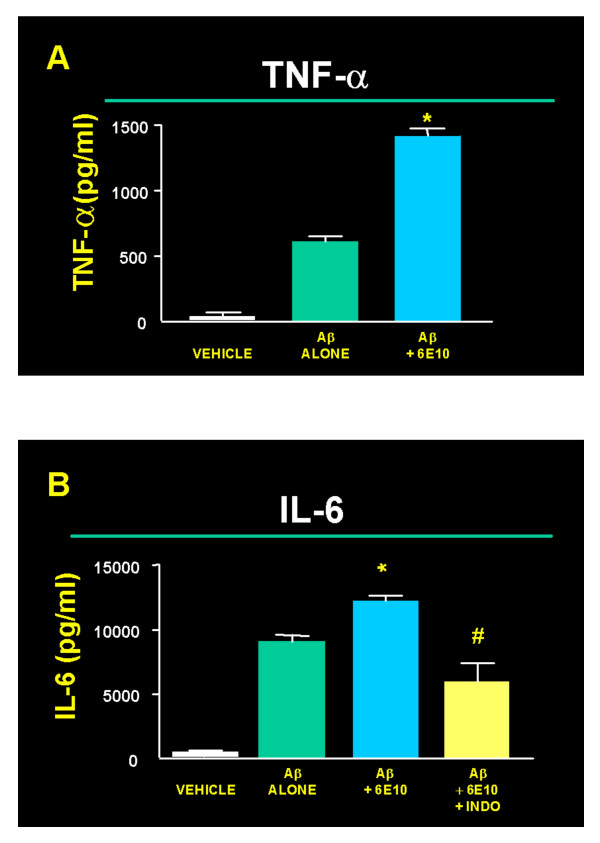
**Effects on microglial TNF-α (A) and IL-6 (B) secretion into the medium in the presence or absence of Aβ, as well as after pretreatment of Aβ with 10 μg/ml anti-Aβ antibody 6E10. **Opsonization with 6E10 significantly enhanced (P < 0.05) (*) TNF-α and IL-6 levels compared to Aβ alone. IL-6 experiments also measured the effect of 1 μg/ml indomethacin on 6E10 exacerbation of cytokine secretion. Indomethacin significantly reduced this effect (P < 0.05) (#).

Consistent with our previous studies covering a wide range of cytokines, chemokines, and inflammatory toxins [12], exposure of microglia to Aβ significantly enhanced secretion of TNF-α (Fig. 5A) and IL-6 (Fig. 5B) compared to cultures that were not exposed to Aβ. Opsonization with 10 μg/ml anti-Aβ antibody 6E10 significantly enhanced Aβ-induced TNF-α (Fig. 5A) and IL-6 secretion (Fig. 5B). Enhancement of IL-6 expression, however, was significantly decreased by indomethacin treatment (Fig. 5B). Cytokine secretion is typically a fairly rapid response that wanes over time. Presumably, cytokine receptive cells then undergo more long-lasting responses such as enhanced chemotactic or phagocytic behaviors. Consistent with this, we observed significant changes in TNF-α and IL-6 levels 4 hours after exposure of microglia to Aβ, but not 3, 6, or 9 days after exposure to Aβ (data not shown).

## Discussion

The present study found that AD microglia in vitro migrate toward Aβ aggregates, attempt to phagocytose the aggregates, and increase their secretion of TNF-α and IL-6 in the process. Opsonization of Aβ aggregates with anti-Aβ antibody 6E10 significantly enhanced these processes. By contrast, the common NSAID indomethacin had no material or statistical effect on microglial migration or phagocytosis, but significantly inhibited the increased IL-6 secretion observed with anti-Aβ opsonization.

The shifts in microglial distributions relative to Aβ spots over time are most parsimoniously explained by chemotactic responses to Aβ. Proliferation of microglia more proximal to Aβ aggregates was not observed and, in fact, BrdU reactivity, a common marker for cell proliferation, was negligible at all distances from the aggregates. Chemokinesis, enhanced but undirected movement of cells, also did not appear to explain the results, since microglial migration exhibited the gradient characteristics of chemotaxis, with progressive increases in the density of microglia at distances more proximal to Aβ aggregates. In addition, microglia are now well established to express receptors that can mediate chemotactic behaviors and that appear to have Aβ as a ligand. These include the macrophage scavenger receptor [16-18], the receptor for advanced glycation endproducts (RAGE) [15], the formyl peptide receptor [19], and others [20,21]. RAGE, in particular, has been shown to help mediate microglial migration to Aβ spots in an in vitro paradigm similar to that used here, and this migration could be inhibited by anti-RAGE Fab fragments [15].

AD microglia in vitro also exhibited behaviors consistent with phagocytosis of Aβ aggregates. Entering the paradigm, the microglia showed little or no Aβ immunoreactivity. After 12 days incubation with Aβ spots, the microglia were highly immunoreactive for Aβ and the spots decreased in size. Aβ spots without microglia remained essentially intact over the same time period. Previous ultrastructural and other studies [3,22,23] have also identified Aβ filaments within microglia in the vicinity of Aβ deposits in AD cortex. Although it remains possible that the intracellular Aβ within microglia in the AD brain may have been produced by the cells [24] rather than phagocytosed from an extracellular deposit, this is clearly not the process observed in the present in vitro studies. We conclude, therefore, that AD microglia in vitro do phagocytose aggregated Aβ deposits. Given the experimental accessibility of the model, it will be of interest in future to evaluate the molecular fate of phagocytosed Aβ in cultured AD microglia.

Exposure to aggregated Aβ also induced significant increases in TNF-α and IL-6 secretion, confirming our previous experiments [12] and those of others [25-27] with TNF-α, IL-6, and a broad range of chemokines, cytokines, and inflammatory toxins such as reactive oxygen/nitrogen species. Pathways for enhancing TNF-α and IL-6 secretion have been demonstrated, including NF-kB and C/EBP transcriptional mechanisms, both of which are enhanced in pathologically-vulnerable regions of the AD brain [28,29].

Opsonization of Aβ spots with anti-Aβ antibody 6E10 significantly enhanced microglial migration to the spots, phagocytosis of the spots, and cytokine secretion. Similar effects of opsonization on microglial migration and phagocytosis have also been reported using anti-Aβ antibodies and an in vitro preparation in which cultured rodent microglia were seeded onto postmortem AD cortex sections laden with Aβ deposits [6]. Soluble Fab fragments containing the Fc region ligand for Fc receptor binding inhibited Aβ removal in this paradigm. These effects are consistent with the classic mechanisms of antibody opsonization of immune targets by antibodies specific to epitopes on the target. Scavenger cells that express receptors to the Fc region of the antibodies are then directed to or become focused at the site where the antibody-bound target resides. Fc receptor activation, in addition, activates scavenger cells, promoting attack and phagocytosis. Recently, scientists at Elan Pharmaceuticals have attempted to harness these mechanisms to enhance Aβ clearance, using immunization with Aβ to drive production of anti-Aβ antibodies for subsequent Aβ opsonization [6,30]. Although there is controversy about the exact site of action of the antibodies (e.g., brain versus peripheral circulation) [6,30,31], this approach does clearly result in significant and sometimes dramatic reductions of Aβ burden in transgenic mouse models [6], as well as the in vitro model tested here, and may also have been effective in human patients receiving Aβ immunization [30].

Unfortunately, however, inflammatory responses are often a two-edged sword. Fc receptor binding is known to enhance the activation and pro-inflammatory secretory responses of scavenger cells that bear Fc receptors, and microglia do express these receptors [6,32]. The increased TNF-α and IL-6 secretion observed in the present experiments after opsonization of Aβ aggregates with a specific anti-Aβ antibody, 6E10, is therefore not unexpected. On activation, microglial cells are, in fact, well established to secrete a wide range of inflammatory mediators that could not only cause damage to neurons and neurites locally, but also, if sufficiently activated, provide signalling to peripheral immune cells to provoke a more generalized and severe response such as that reported in several Aβ-immunized patients who experienced lethal adverse reactions [30].

The vast majority of NSAIDs in use today are based on the principle of cyclooxygenase inhibition, and cyclooxygenase inhibition, in turn, is well established to downregulate a wide range of acute phase reactants. Interestingly, however, mechanisms for chemotaxis to and phagocytosis of an inflammatory target are not necessarily cyclooxygenase dependent. In a survey, for example, of the first 100 publications retrieved from PubMed using the search phrase "indomethacin AND chemotaxis", the majority of studies found no effect of indomethacin on chemotaxis, and some of the papers actually reported enhanced chemotaxis after indomethacin exposure. Such findings have been suggested to explain why physicians commonly prescribe NSAIDs to control fever and other secondary inflammatory responses without being unduly concerned about hampering immune-mediated removal of the fever-inducing agent. Similarly, in the present experiments indomethacin had no material or statistically significant effect on microglial chemotaxis to or phagocytosis of Aβ aggregates, but did significantly inhibit the exacerbated IL-6 response under opsonized conditions. Although it is never certain that in vitro results will fully apply to the in vivo state, these results suggest that indomethacin or an NSAID like it might be a useful adjunct to Aβ immunization strategies.

## Competing interests

JR is a co-inventor on an issued United States patent covering use of nonsteroidal anti-inflammatory drugs as a treatment for Alzheimer's disease. All other authors declare that they have no competing interests.

## Authors' contributions

JR conceived and designed the experiments, performed all data analysis, and wrote the manuscript. RS supervised and took part in all experiments. CJK performed the chemotaxis, phagocytosis, and cytokine experiments. DM, BL, and AG prepared cultures and performed histochemistry and immunocytochemistry.
